# Editorial: Early prediction of CNS problems by combined ocular markers (and vice versa)

**DOI:** 10.3389/fnint.2024.1394254

**Published:** 2024-03-14

**Authors:** Reza Rastmanesh

**Affiliations:** Biomedical Sciences Department, American Physical Society, College Park, MD, United States

**Keywords:** CNS, visual system, Alzheimer, schizophrenia, dementia, glaucoma, cognition, aging

Visual impairment (VI) has been proposed as one of the early symptoms of dementia. Extensive investigations have reported similar neuronal and microvascular alterations in the eye and brain in patients with cognitive impairment (CIM) or dementia. Many other studies have examined the associations between different ocular diseases and the causes of CIM.

VI and CIM share many risk factors such as vascular and medical co-morbidities, age, physical inactivity, etc.; and also common outcomes such as falls, functional decline, deterioration in quality of life, and mortality. Many types of VI and CIM now share common factors and pathophysiologic processes. For instance, age-related macular degeneration has been associated with Alzheimer's disease and Parkinson's disease.

The results of a longitudinal study and a well-conducted meta-analysis have specifically shown the existence of a bidirectional relationship between VI and CIM. These studies, however, focused only on visual acuity (using Early Treatment Diabetic Retinopathy Study charts) and cognitive status [using the Mini-Mental State Examination (MMSE)] and did not include other components of vision (such as contrast sensitivity, stereo acuity, color vision, and visual hallucinations).

Since the brain and eye are neural tissues derived from the same embryonic germ layer, the coincidence of developmental anomalies in these organs is not surprising. Recently, some specific aquaporins (AQPs) have been shown to have regional functions in the development of refractive index in the zebrafish eye lens. In humans, certain refractive errors are associated with some specific CIMs. Since in principle, there are no qualitative differences between the AQPs of the visual system and the central nervous system, early detection of patterns of these and similar easily accessible/measurable ocular markers/biomarkers and indices would provide next-generation biotechnology for the early diagnosis of brain diseases (and vice versa), with high sensitivity/precision/accuracy and cost-effectiveness.

If bidirectional relationships are present between different types of VI and different types of CIM, and if such associations could be determined at an early stage using easily measurable biomarkers, it would provide unique opportunities for the development of early detection and management of risk factors for both VI and CIM in all age groups.

In this Research Topic of Frontiers in Medicine (section Ophthalmology), Frontiers in Aging Neuroscience (section Cellular and Molecular Mechanisms of Brain-aging), and Frontiers in Integrative Neuroscience, new evidence has been gathered on the state of the art of Early Prediction of CNS Problems by Combined Ocular Markers (and vice versa) and its implications. While still an emerging discipline in the ophthalmology and neuroscience curriculums, these studies offer valuable information in the form of systematic reviews/meta-analyses, preclinical cell studies, hypotheses and clinical studies in this field. In their clinical study, Tarrit et al. found no evidence of a slower rate of adaptation during the early adaptation phase, and no evidence of greater variance in saccade amplitudes in autism spectrum disorder (ASD) in either children or adults on the spectrum. This is a very important finding because previous studies reported in the literature have yielded mixed results regarding the ability of individuals with ASD to adapt saccade amplitudes in response to imposed visual errors. There was also no evidence of a slower rate of adaptation in ASD either in children or adults.

Acupuncture studies represent novel therapeutic avenues in ophthalmological neuroscience. Acupuncture has certain effects on improving visual function in myopia, but its neural mechanism is unclear. Su et al. studied the effect of acupuncture on visual function and electroencephalography (EEG) microstates in myopia. The authors applied acupuncture to the right Taiyang acupoint of myopic patients to analyze the effects of acupuncture on visual function and electroencephalographic activity and to investigate the correlation between improvements in visual function and changes in the brain. The subjects' contrast sensitivity (CS) was examined before and after acupuncture, and EEG data were recorded throughout the acupuncture process. They found that compared to before acupuncture, the CS of both eyes of myopic patients was increased at each spatial frequency after acupuncture; compared to the resting state, the contribution of microstate C was decreased during the post-acupuncture state, and the transition probability between microstate A and microstate C was reduced; in addition, the contribution of microstate C was negatively correlated with CS at both 12 and 18 cpd.

Al-Nosairy et al. suggested functional and structural readouts for early detection of retinal involvement in multiple sclerosis. They differentially assessed photoreceptor/bipolar cell (distal retina) and retinal ganglion cell (RGC, proximal retina) function in addition to structural assessment (OCT). They also compared two multifocal electroretinography-based approaches, i.e., the multifocal pattern electroretinogram (mfPERG) and the multifocal electroretinogram recording the photopic negative response (mfERG_*PhNR*_). For structural assessment, peripapillary retinal nerve fiber layer (pRNFL) thickness and macular scans were used to calculate the outer nuclear layer (ONL) and macular ganglion cell inner plexiform layer (GCIPL) thickness. One eye per subject was randomly selected. The authors demonstrated that while structural damage was evident mainly for a history of optic neuritis, functional measures were the only retinal readouts of MS-related retinal damage that were independent of optic neuritis, as observed for NON. These results indicate that MS-related inflammatory processes in the retina precede optic neuritis. The authors particularly emphasized the importance of retinal electrophysiology in MS diagnostics and its potential as a sensitive biomarker for follow-up in innovative interventions.

As for preclinical cell studies, Pediconi et al. examined the retinal cell layers of sporadic ALS patients in postmortem retinal slices by immunofluorescence analysis. They evaluated the presence of cytoplasmic TDP-43 and SQSTM1/p62 aggregates, activation of the apoptotic pathway, and microglia and astrocyte reactivity. The authors documented an increase in mislocalized TDP-43, SQSTM1/p62 aggregates, cleaved caspase-3 activation, and microglial density in the retinal ganglion cell layer of ALS patients, suggesting that retinal changes may be used as an additional diagnostic tool for ALS. They also suggested that *in vivo* retinal biomarkers as an additional diagnostic tool for ALS may provide an opportunity to longitudinally monitor individuals and therapies over time in a non-invasive and cost-effective manner.

Tsitsi et al. investigated differences in pupil light reflex (PLR) parameters, measured by an eye tracker, between patients with Parkinson's disease (PD), with and without signs of dysautonomia, and healthy controls (HC). Their results support previous observations of a defective PLR in PD, assessed by eye tracker, and suggest a possible association with autonomic dysfunction.

Lopergolo et al. hypothesized that autosomal recessive cerebellar ataxias (ARCAs) can be used as a diagnostic classification approach based on ocular features. The authors discussed the results of clinical and eye-tracking oculomotor examinations, the OCT findings and some advances in computer science in ARCAs thus providing evidence to support the identification of robust eye parameters as possible markers for ARCAs.

Lastly, in a systematic review and meta-analysis, Zhang et al. investigated whether cataract extraction lowers the risk of all-cause dementia. The authors documented that cataract surgery was associated with a lower incidence of all-cause dementia and Alzheimer's disease. Cataracts are a reversible visual impairment. Cataract surgery may be a protective factor against the onset of all-cause dementia and may reduce the economic and family burden of all-cause dementia worldwide. However, given the limited number of included studies, their findings need to be interpreted with caution.

## Author contributions

RR: Writing – original draft, Writing – review & editing.

